# MiR-107 and MiR-185 Can Induce Cell Cycle Arrest in Human Non Small Cell Lung Cancer Cell Lines

**DOI:** 10.1371/journal.pone.0006677

**Published:** 2009-08-18

**Authors:** Yukari Takahashi, Alistair R. R. Forrest, Emi Maeno, Takehiro Hashimoto, Carsten O. Daub, Jun Yasuda

**Affiliations:** 1 RNA Function Research Team, Omics Science Center, RIKEN Yokohama Institute, Yokohama, Japan; 2 LSA Technology Development Group, Omics Science Center, RIKEN Yokohama Institute, Yokohama, Japan; 3 LSA Bioinformatics Core Facility, Omics Science Center, RIKEN Yokohama Institute, Yokohama, Japan; 4 The Eskitis Institute for Cell and Molecular Therapies, Griffith University, Nathan, Queensland, Australia; Roswell Park Cancer Institute, United States of America

## Abstract

**Background:**

MicroRNAs (miRNAs) are short single stranded noncoding RNAs that suppress gene expression through either translational repression or degradation of target mRNAs. The annealing between messenger RNAs and 5′ seed region of miRNAs is believed to be essential for the specific suppression of target gene expression. One miRNA can have several hundred different targets in a cell. Rapidly accumulating evidence suggests that many miRNAs are involved in cell cycle regulation and consequentially play critical roles in carcinogenesis.

**Methodology/Principal Findings:**

Introduction of synthetic miR-107 or miR-185 suppressed growth of the human non-small cell lung cancer cell lines. Flow cytometry analysis revealed these miRNAs induce a G1 cell cycle arrest in H1299 cells and the suppression of cell cycle progression is stronger than that by Let-7 miRNA. By the gene expression analyses with oligonucleotide microarrays, we find hundreds of genes are affected by transfection of these miRNAs. Using miRNA-target prediction analyses and the array data, we listed up a set of likely targets of miR-107 and miR-185 for G1 cell cycle arrest and validate a subset of them using real-time RT-PCR and immunoblotting for CDK6.

**Conclusions/Significance:**

We identified new cell cycle regulating miRNAs, miR-107 and miR-185, localized in frequently altered chromosomal regions in human lung cancers. Especially for miR-107, a large number of down-regulated genes are annotated with the gene ontology term ‘cell cycle’. Our results suggest that these miRNAs may contribute to regulate cell cycle in human malignant tumors.

## Introduction

miRNAs are 19 to 23-base long single stranded RNAs that play critical roles in biological processes [1]. The nucleotide sequences of miRNAs are often evolutionally conserved among multicellular organisms [2]. The miRNAs are expressed as hairpin shaped double stranded pre-miRNAs and sequential processing by different RNase III enzymes, Drosha and Dicer, generates mature miRNA [3].The mature miRNA binds with a set of proteins, including Agonaute, to form a miRNA induced silencing complex (miRISC). The miRISC is believed to make a complex with target messenger RNAs and post-transcriptionally suppresses the expression of the target genes. The mechanism of action of miRISC is still controversial [4], however, there is a general consensus that majority of target messenger RNAs have binding sites for the miRNAs in the 3′ untranslated regions. From second to eighth bases of 5′ end sequence of miRNA is called seed sequence and is believed to be essential for the recognition of the target messenger RNAs by miRNAs.

It has become evident that some miRNAs play critical roles in the cell cycle regulation in cooperation with the oncogenes or tumor suppressor genes (see review [5], [6]). One example of cell cycle regulating miRNA is the *let-7*(for *hsa-let-7a*, MIMAT0000062). The introduction of synthetic pre-let-7 causes the cell cycle arrest in lung cancer cells [7]. Many miRNAs are known to downregulate cell cycle related genes. The miR-17∼92 cluster was identified as the downstream of the *MYC* oncogene [8] and downregulate E2F transcription factors which are well-known mediators of cell cycle progression [9].Another important tumor related gene, the *TP53*, induce the expression of miR-34 family members and overexpression of miR-34 caused the cell cycle arrest at the G1 phase [10]–[16].

Here we report potential cell cycle regulating miRNAs during the search of cancer related miRNAs in human lung cancer cells. In this study we revisit a set of genomic regions identified by Zhao *et al*. that are amplified or deleted in human lung cancers [17]. During the study, we found that *miR-107* (MIMAT0000104) and *miR-185* (MIMAT0000455) suppress proliferation in lung adenocarcinoma cell lines and induce cell cycle arrest at the G1 phase of the cell cycle. We attempted to characterize downstream target messenger RNAs of these miRNAs by the use of microarray profiling with gene ontology analyses and TargetScan predictions [18].

## Results

### Expression of miR-31, 107, and 185 in human tissue collection including lung cancer tissue and cell lines

From the regions identified by Zhao *et al*. [17], we found 13 and 26 annotated miRNAs in the homozygously deleted and amplified regions respectively (supplementary table S1). Because many of the cancer related genes contribute to malignant transformation in wide spectrum of cell types, we prioritized miRNAs that have been implicated in other adult-onset human cancers. Then we chose three miRNAs : miR-107, miR-185, and miR-31 (MIMAT0000089) [19]–[22]. We added the let-7a for cell growth and cell cycle control because the miRNA can suppress cell growth in the lung cancer cell lines [23] and induce cell cycle arrest in the HepG2 cell line [7].

**Figure 1:**
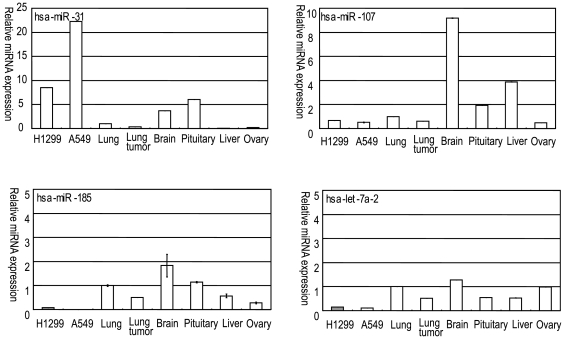
Expression of candidate miRNAs in normal tissues and lung cancer. miRNA expression levels were measured by miRNA TaqMan qRT-PCR in normal lung, brain, pituitary, liver and ovary tissues, a lung tumor sample and also in the lung tumor cell lines, H1299 and A549. The vertical axis indicates the relative expression of each miRNA normalized with that of RNU44.

Using Taq-Man quantitative RT-PCR technology, we measured the expression of the four miRNAs in the human lung cancer cell lines A549 and H1299 and across a panel of commercially available RNAs from normal tissue and lung cancer samples (Fig. 1). Most of the miRNAs showed relatively ubiquitous expression among healthy tissues (Fig. 1). It is interesting that expression of the analyzed miRNAs (miR-107, 185, and let-7a) were lower in the lung tumor and lung cancer cell lines than in normal lung. The miR-31 was highly expressed in the lung cancer cell lines.

### Growth suppression and cell cycle arrest by over-expression of candidate miRNAs in human lung cancer cell lines

**Figure 2:**
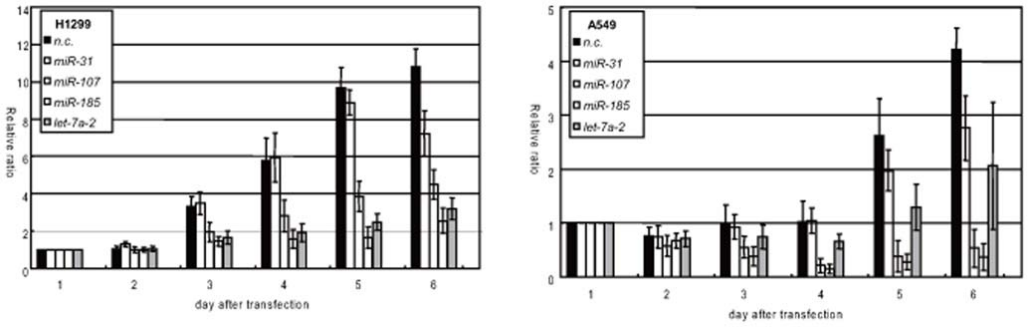
Overexpression of miR-107 and miR-185 causes growth suppression and induces G1 cell cycle arrest. Growth suppression effect of miRNA candidate transfections on H1299 (left panel) and A549 (right panel) as measured by MTT assay. The vertical axis indicates the relative ratio of the A450 nm: that of day 0 of each cell as 1. Note miR-107 and miR-185 suppresses proliferation in both cell lines.

The effect of these miRNAs on proliferation was tested by MTT assay with pre-miRNA transfected H1299 and A549 cells. Transfection of hsa-miR-107 and hsa-miR-185 dramatically reduced cell proliferation in both cell lines (Fig. 2). In the case of H1299 cells, the let-7a miRNA showed significantly reduced proliferation while the effects were less obvious in A549 cells. The extent of growth suppression of A549 by let-7a is similar to that of reported [7]. The miR-31 showed slight suppression of cell growth but the suppression levels were not statistically significant at many time points for both of the cells (Fig. 2).

**Figure 3:**
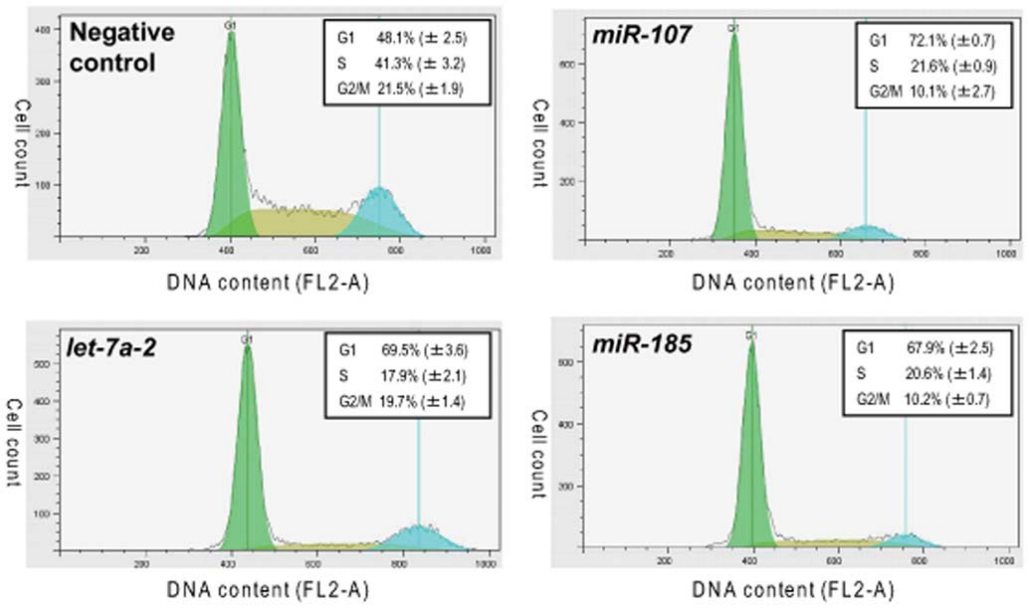
Effect of miR-107, miR-185 and let-7a over-expression on cell cycle profile in H1299 cells. Histograms of DNA contents obtained by FACS analysis are shown. The percentages of each cell cycle stages are shown in the inset of the histograms. There was no gate applied to the events so that there was no obvious accumulation of sub-G1 populations in all the experiments.

DNA content analysis by flow cytometry revealed transfection of hsa-miR-107 and hsa-miR-185 induced a significant increase in the percentage of cells at the G1 phase of the cell cycle, to similar levels as a let-7a control while a scrambled negative control did not (Fig. 3). We did not observe either any apparent increase of the sub-G1 population in the flow cytometry or any apoptosis-related morphological changes, such as nuclear blebbing and condensation, under the phase contrast microscope (data not shown). This suggests that growth suppression induced by hsa-miR-107 and hsa-miR-185 transfection was caused by induction of G1 arrest rather than apoptosis.

### Identification of candidate target mRNAs of the miRNAs by gene profiling analysis

The microarray profiling was done to determine the global changes in mRNA expression levels in H1299 cells transfected with growth suppressive miRNAs compared to a negative control. In the hsa-miR-107, hsa-miR-185 and hsa-let-7a transfected cells there were 561, 646 and 812 transcripts down-regulated and 608, 698 and 949 upregulated by 1.5 fold or greater, respectively. Gene Ontology analysis was carried out on the genes down-regulated in the transfectans. Table 1 lists the most significantly enriched GO terms for the down-regulated genes with each miRNA. The top five terms in genes down-regulated by hsa-miR-107 were all cell cycle related (table 1). Down-regulated genes with the let-7a were mainly involved in rRNA metabolism, ribosome biogenesis, and the M phase of the cell cycle. Finally, the down-regulated genes with hsa-miR-185 showed no enrichment for cell cycle related terms, instead of the terms related to the development and differentiation were prominent. In addition the 127 and 33 genes commonly down-regulated and upregulated respectively by both miRNAs showed no gene ontology enrichments, suggesting that these miRNAs induce cell cycle arrest with different signaling pathways (tables 2, 3, and data not shown).

**Table 1:** Gene ontology terms enriched of genes down-regulated by miR-107, miR-185 and let-7a transfection.

GO category	GO terms (# of genes)	# of genes affected	P-value
**mir107**			
GO:0022403	cell cycle phase (304)	135	7.66E-14
GO:0022402	cell cycle process (519)	203	1.58E-13
GO:0007049	cell cycle (635)	238	1.73E-13
GO:0000279	M phase (248)	114	1.82E-13
GO:0051301	cell division (197)	95	6.04E-13
GO:0000278	mitotic cell cycle (279)	119	6.71E-11
GO:0000087	M phase of mitotic cell cycle (203)	93	9.18E-11
GO:0007067	mitosis (201)	92	1.18E-10
GO:0006259	DNA metabolic process (665)	218	0.0000106
GO:0006260	DNA replication (190)	77	0.0000235
GO:0006974	response to DNA damage stimulus (283)	105	0.0000428
GO:0006281	DNA repair (232)	88	0.000126
GO:0007059	chromosome segregation (55)	28	0.00108
GO:0000070	mitotic sister chromatid segregation (26)	16	0.00341
GO:0006270	DNA replication initiation (26)	16	0.00341
GO:0000819	sister chromatid segregation (27)	16	0.00732
**mir185**			
GO:0032501	multicellular organismal process (2482)	695	1.26E-14
GO:0007275	multicellular organismal development (1739)	496	4.76E-11
GO:0048513	organ development (900)	283	7.98E-11
GO:0048731	system development (1265)	376	7.98E-11
GO:0048856	anatomical structure development (1576)	453	8E-11
GO:0032502	developmental process (2503)	664	8.08E-09
GO:0009653	anatomical structure morphogenesis (831)	248	0.000000877
GO:0009888	tissue development (234)	88	0.000000914
GO:0030154	cell differentiation (1404)	387	0.00000199
GO:0048869	cellular developmental process (1404)	387	0.00000199
GO:0007154	cell communication (2838)	722	0.0000032
GO:0007166	cell surface receptor linked signal transduction (1061)	300	0.00000713
GO:0007165	signal transduction (2569)	651	0.0000395
GO:0009887	organ morphogenesis (301)	102	0.0000395
GO:0001568	blood vessel development (139)	54	0.000197
GO:0001944	vasculature development (140)	54	0.000252
GO:0008277	regulation of G-protein coupled receptor protein signaling pathway (26)	16	0.000365
GO:0048514	blood vessel morphogenesis (125)	49	0.000365
GO:0048646	anatomical structure formation (128)	50	0.000365
GO:0015031	protein transport (616)	85	0.000404
GO:0008104	protein localization (684)	98	0.00055
GO:0033036	macromolecule localization (722)	105	0.00055
GO:0009611	response to wounding (295)	96	0.000577
GO:0006412	translation (455)	58	0.000646
GO:0007186	G-protein coupled receptor protein signaling pathway (531)	157	0.000646
GO:0044237	cellular metabolic process (6365)	1257	0.000646
GO:0006952	defense response (350)	110	0.000684
GO:0045184	establishment of protein localization (650)	93	0.000684
GO:0048771	tissue remodeling (76)	33	0.000684
GO:0001501	skeletal development (163)	59	0.000703
**let7a**			
GO:0042254	ribosome biogenesis (85)	60	2.32E-16
GO:0022613	ribonucleoprotein complex biogenesis and assembly (177)	98	6.22E-15
GO:0006364	rRNA processing (60)	41	5.53E-10
GO:0010467	gene expression (2934)	929	7.63E-10
GO:0016072	rRNA metabolic process (63)	42	7.63E-10
GO:0006139	nucleobase, nucleoside, nucleotide and nucleic acid metabolic process (3216)	991	0.000000141
GO:0006396	RNA processing (419)	167	0.000000191
GO:0016070	RNA metabolic process (2479)	774	0.00000174
GO:0043170	macromolecule metabolic process (5608)	1634	0.00000248
GO:0044237	cellular metabolic process (6395)	1830	0.0000105
GO:0044238	primary metabolic process (6396)	1834	0.000024
GO:0043283	biopolymer metabolic process (4287)	1256	0.000159
GO:0007154	cell communication (2838)	648	0.000212
GO:0006412	translation (455)	163	0.00124
GO:0044249	cellular biosynthetic process (860)	285	0.00124
GO:0032501	multicellular organismal process (2482)	570	0.00255
GO:0009451	RNA modification (31)	19	0.00499
GO:0006399	tRNA metabolic process (111)	49	0.00634
GO:0007267	cell-cell signaling (465)	85	0.00923

**Table 2:** List of commonly downregulated cell cycle related genes by transfection of different miRNAs.

entrez symbol	Fold change in expression		
	mir-107	mir-185	let-7
SMG6	0.130749	0.126058	0.111471
FUNDC2	0.570185	0.523724	0.492232
MUC20	0.64695	0.499866	0.323235
BCL2L11	0.736578	0.41652	NS
CCNE1	0.376009	0.512158	NS
CDK6	0.565894	0.705571	NS
RASSF5	0.423999	0.44275	NS
RUNX3	0.653897	0.731732	NS
VEGFA	0.690304	0.6205	NS
XRCC3	0.723088	0.537486	NS
TUBGCP3	0.365103	0.607218	NS
TPD52	0.36629	0.639004	NS
RAB1B	0.470178	0.397512	NS
MAP9	0.502884	0.719742	NS
RAPGEF1	0.515269	0.726705	NS
CDK5R1	0.538231	0.638624	NS

NS: Not suppressed.

**Table 3:** List of commonly downregulated oncogenes by transfection of different miRNAs.

entrez symbol	Fold change in expression		
	mir-107	mir-185	let-7
FUNDC2	0.5701851	0.5237243	0.49223238
MUC20	0.64695007	0.49986598	0.32323453
BCL2L11	0.73657805	0.41651997	NS
VEGFA	0.6903044	0.62050027	NS
TPD52	0.36629018	0.6390038	NS
RAB1B	0.47017783	0.3975123	NS
RAPGEF1	0.51526946	0.72670466	NS
PIM1	0.66107285	0.48559082	NS
RAB35	0.71937436	0.30117804	NS
HMGA2	NS	0.4689389	0.052681383
FGF5	NS	0.62565315	0.3222275
PATZ1	NS	0.6462347	0.5966134
CCND2	0.7438488	NS	0.523334
CBL	0.41297674	NS	0.4365899
RGPD5	0.63766026	NS	0.7261504
ABL2	0.7186717	NS	0.67202175

NS: Not suppressed.

**Figure 4:**
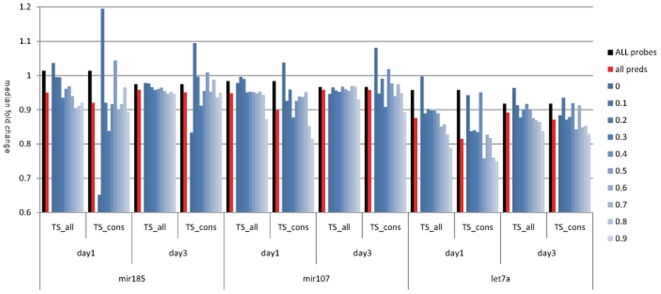
Plot of median signal of TargetScan predicted targets at different thresholds. Target scan predictions were extracted for miRNAs 185, 107 and let7a. Median expression signal is shown only for genes considered as detected by the Agilent software. Y-axis indicates the median fold change for sets of predicted microRNA target genes at different thresholds, compared to all genes on the microarray (shown in black). In all cases the median signal of predicted targets is lower than that observed if all probes are used. When the experiment is extended out to three days, we observe less of an effect, suggesting direct targets are more affected within the first day.

We compared the miRNA target predictions with the TargetScan software [18] for these miRNAs to the genes down-regulated in our expression profiling datasets. For both conserved and non-conserved sites, we found the median fold change of predicted targets was consistently lower than that of all genes detected in the arrays (Fig. 4). There is a trend for more strongly predicted targets to be more down-regulated than weak predicted targets. Similarly, the conserved targets tend to be more down-regulated than all predicted targets (Fig. 4).

We then matched these computer-predicted targets to the genes down-regulated more than 0.75 fold at the RNA level to narrow down the potential targets of these miRNAs. We found many of these potential targets were annotated with the terms “cell cycle” in Entrez Gene annotations (table 2) suggesting that these three miRNAs may directly regulate the cell cycle progression through these genes. Interestingly, in our hand, the let-7 did not suppress the expression of the CDK6 (NM_001145306 ) mRNA, which is suppressed by the overexpression of let-7 in the previous study [7]. Table 3 indicates that distinct sets of known oncogenes are downregulated by these miRNAs.

**Figure 5:**
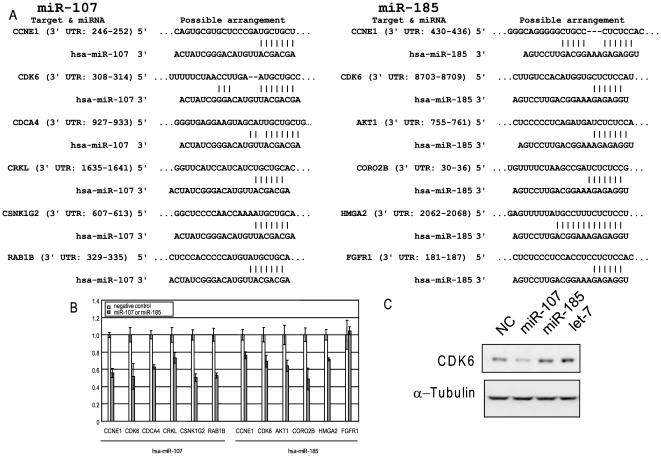
Confirmation of mRNA down-regulation by qRTPCR for predicted targets. A) Representative nucleotide sequence matches between possible target genes and miRNAs. The numbers in parenthesis indicates the positions of target nucleotides from the stop codon. Only matched nucleotides with miRNA seed sequences are indicated with the vertical lines. B) The quantitative RT-PCR analyses of potential targets of miR-107 (CCNE1, CDK6, CDCA4, RAB1B and CRKL) and miR-185 (CCNE1, CDK6, AKT1, HMGA2, CORO2B) are shown. The vertical axis indicates the relative expression ratio of each gene normalized with that of GAPDH. C) Western Blot showing down-regulation of CDK6 protein by miR-107.

From these lists and other list describing candidate miRNA targets annotated with the terms cell cycle, lung cancer, oncogene or tumor suppressor in Entrez Gene annotations (supplementary table S2), we chose a subset of transcripts for validation by qRT-PCR by the comparison with the target lists provided by TargetScan [18] and PicTar [24] software (Fig. 5A). For miR-107, we confirmed mRNA down-regulation of *CCNE1* (NM_001238), *CDK6*, *CDCA4* (NM_017955.3), *RAB1B* (NM_030981.2) and *CRKL* (NM_005207.3), and for miR-185, we confirmed down-regulation of *CCNE1*, *CDK6*, *AKT1* (NM_001014431.1), *HMGA2* (NM_003483.4) and *CORO2B* (NM_006091.3) (Fig. 5B). We note that both miR-107 and miR-185 transfection caused down-regulation of cyclin E1 (CCNE1) and cyclin dependent kinase 6 (CDK6) mRNA levels although the suppression level of CDK6 by miR-185 is modest (Fig. 5B). We then confirmed by western blotting that CDK6 protein levels are also down-regulated by miR-107, whereas CDK6 expression was relatively unchanged by miR-185 (Fig. 5C). Because the suppression level of CDK6 mRNA expression by miR-185 is very modest, the subsequent decrease of CDK6 protein expression at the time point of observation (24 hours after transfection) may be too little to be observed the conventional immunoblottings.

## Discussion

We happened to find that miR-107 and miR-185 can suppress cell proliferation in two lung cancer cell lines and induced a G1 arrest of the cell cycle. The extent of growth suppression by these miRNAs is similar to that by the tumor suppressive miRNA, let-7. Gene expression profiling analysis with the transfection of these miRNAs indicated that only miR-107 showed significant enrichment of cell cycle regulators for the downstream effectors. On the other hand, miR-185 did not significantly repress cell cycle regulator as well as let-7, a known cell cycle regulating miRNA [6]. The miR-185, however, could suppress the mRNA expression of cell cycle regulating genes such as CDK6 and AKT1.

The commonly regulated gene sets by all three growth suppressive miRNAs are not so many and not so strongly related to the cell cycle regulation (Table 2). These results suggested that the three miRNAs regulate distinct cellular signaling pathways. Since miRNA has a wide range of targets in a cell (i.e. less specific) and since the extent of suppression of the target expression by miRNA is generally moderate, the function of miRNAs should be considered as the “fine tuning” of gene expression in mammalian cells [25]. The accumulation of these small regulatory effects may cause the significant biological reactions in the cells [5].On the other hand, a few potential target molecules such as CCNE1 and CDK6 may be critical for cell cycle regulation by these miRNAs. For example, reduction of CCNE1 with siRNA causes cell cycle arrest in liver cancer cell lines [26]. In the case of CDK6, reduction of CDK6 by siRNA caused prolonged S-phase in human embryonic stem cells [27]. In general, the importance of miRNA in cell cycle regulation is quite reasonable because miRNAs are supposed to be key molecules for induction of cell differentiation, which accompanies with cell cycle arrest in many cases.

In terms of miR-107, other evidence supports a role for this miRNA in G1 arrest and growth suppression. miR-107 shares 7 of the 8 bases of its seed sequence with the miR-16 family of miRNAs, which induce G1 arrest by targeting multiple cyclins and cell cycle regulators, including CDK6 which we confirmed as a miR-107 target [28]. Furthermore, a previous study found that synthetic inhibitors for miR-107 increase proliferation of A549 cells, but do not effect HeLa cells [29] suggesting miR-107 may indeed play a lung specific role in reducing proliferation. Interestingly, the miR-107 showed overexpressions in pancreatic cancers suggesting this miRNA has some positive role in pancreatic carcinogenesis [21]. On the other hand, during the preparation of this manuscript, Lee et al. reported that demethylation and deacetylation treatments to human pancreatic cancer cell lines induced the overexpression of miR-107 and the overexpression of miR-107 suppressed cell growth and the expression of the CDK6 in the human pancreatic cancer cell lines [30]. The latter study is compatible to our data in terms of CDK6 as a candidate downstream target of miR-107. It is interesting whether this miRNA did have any specific cellular functions in the cells rather than cell cycle regulation. Safdar *et al*. suggested that miR-107 has been induced in exercised mice quadriceps muscles [31]. According to the review by Wilfred et al., the miR-107 and its paralog, miR-103, may function in the regulation of cellular metabolism [32]. It may be interesting possibility that these miRNAs regulate the fundamental cellular functions such as metabolism or cell cycle progression rather than the specification of cell differentiation.

The mechanism of cell cycle arrest by miR-185 is not clear. The number of cell cycle regulators in the downstream suppressed genes is much lower by miR-185 than by miR-107. One group of scientists suggested that this miRNA is overexpressed in bladder cancer [20]. In the other paper, Choong reported that miR-185 have strong positive correlation to the appearance of erythroid surface antigens (CD71, CD36, and CD235a) in human umbilical cord blood cells stimulated with growth factors and induced erythroid differentiation [33]. Generally speaking, the induction of cell differentiation usually couples with the suppression of cell cycle progression. Considering the miRNA functions in other metazoans, many of the miRNAs inducible with cell differentiation might have some cell cycle suppressive functions. The miRNA should have other biological functions in different cell types. It is therefore interesting to investigate whether miR-185 has any differentiating functions in lung cancer cells. Another important question is that miR-185 showed growth suppressive functions (figures 2, 3) and decrease of expression in lung cancer cells (figure 1) even though the miRNA is localized in a chromosomal region amplified in two lung cancer cell lines ([17] and supplementary table S1). These results are counter-intuitive. It is possible, however, that the tumor suppressors can be localized in a region showing chromosomal amplification in tumor cells. One example is a potential tumor suppressive gene, *GSDMA*(NM_178171.4) [34], is localized in a chromosomal region amplified in gastric cancer cells [35]. This gene is downregulated in the human gastric cancers even though the gene showed amplification in tumor cells [35]. In the case of the miR-185, epigenetic silencing of the miRNA might occur prior to the gene amplification of the chromosome 22q21.1 region. Further investigation clearly needs to address these questions thoroughly.

Finally, we report that new candidate miRNAs which can regulate cell cycle progression in human non-small cell lung cancer cell lines. It is still an open question that whether any somatic genetic alterations can cause the suppression of these miRNAs in human lung cancer or any other malignant tumors. Further characterization of the genomic loci of these miRNAs is necessary to make the issue clear.

## Methods

### Extraction of miRNA position

Annotated miRNA loci were extracted from regions of chromosomal gain and loss identified by Zhao *et al*. [17] in a large panel of human lung carcinomas using SNP arrays. The Hg16 co-ordinates were converted to their Hg18 using equivalents using the UCSC Lift-Over tool (http://genome.ucsc.edu).

### Cell lines

Human lung cancer cell lines, H1299 and A549, were purchased from ATCC. The cell lines were grown in DMEM containing 10% heat-inactivated fetal bovine serum, 100 µg/ml of penicillin/streptomycin and 292 µg/ml of L-glutamine (Invitrogen, Carlesbad, CA, USA).

### RNA preparations and quantification of RNAs using real-time PCR

All the realtime PCR was performed with StepOnePlus Realtime PCR system (Applied Biosystems, Foster City, CA, USA) in quadruplicate. Total RNA was extracted from H1299 and A549 cells with the *mir*Vana miRNA isolation kit (Ambion, Austin, TX, USA). Total RNAs of human tissues were purchased from BioChain Institute, Inc. (Hayward, CA, USA; Catalog numbers: R1234152-50, R1235152-50, R1234035-50, R1234068-10, R1234149-50, R1234183-50). For quantification of miRNAs, 100 ng of total RNA was analyzed with TaqMan MicroRNA Reverse Transcription (RT) Kit (Applied Biosystems) with RNU44 as loading control. For quantification of mRNAs, 500 ng of total RNA was reverse-transcribed using the PrimeScript II RT Enzyme (Takara Bio, Inc., Shiga, Japan) and PCR was performed with SYBR premix Ex Taq (Perfect Real Time: Takara Bio, Inc.) with GAPDH as loading control. All the real-time PCR analysis were done in triplicate.

### miRNA transfection

Synthetic pre-miRNAs and nonspecific negative control (miRIDIAN microRNA Mimic Negative Control#1) were purchased from Dharmacon, Inc. (Lafayette, CO, USA). The pre-miRNAs were transfected at a final concentration of 10 nM with Lipofectamine2000 (Invitrogen), and medium changed 24 hours after transfection.

### Cell growth measurement

Cells were plated in 96-well plates and incubated at 37°C in a 5% CO_2_ incubator. Cell viability was evaluated by MTT assay using the Cell counting kit-8 (DOJINDO, Kumamoto, Japan), according to the manufacturer's protocol. After 1 hour incubation with the media containing tetrazolium compound, the absorbance at 450 nm was detected for 0.1 sec with Arvo MX 1420 (Perkin Elmer, Waltham, MA, USA).

### Cell cycle analysis

Cells were harvested after 72 hours and fixed in 70% ice cold ethanol and followed by RNAse A treatment, stained with 50 µg/ml of Propidium Iodide for DNA content analysis by flow cytometry analysis on a FACS Calibur system (Becton Dickinson, Franklin, NJ, USA). The data were collected and processed using the FlowJo FACS analysis software (Tree Star, Inc., Ashland, OR, USA).

### Expression profiling using Agilent Gene expression array

The cRNA probe was generated from total RNA (500 ng) with the Low RNA Input linear amplification & Labeling kit (Agilent Technologies, Santa Clara, CA, USA). Cy3-labeled cRNA (1.65 µg) was then fragmented and relative expression was measured by hybridization to 4×44K whole human oligo microarray (Agilent). Feature Extraction ver. 9.1 software (Agilent) was used to analyze the image of microarray. The microarray analyses were performed in duplicate. All microarray data reported in the manuscript is described in accordance with MIAME guidelines and the data has been deposited in CIBEX (Center for Information Biology gene Expression) database [36] at Center for Information Biology and DNA Data Bank of Japan (DDBJ), National Institute of Genetics (Mishima, Japan). The accession number for the dataset is CBX79.

### Microarray and Gene ontology analysis

Microarray data was visualized and normalized using Genespring GX 7.3 software (Agilent). Values below 0.01 were set to 0.01. For each chip each measurement was divided by the 50th percentile of all measurements for that chip. Then for each probe the measurements were normalized to the negative control measurements. Because of the limitation of the resources, the statistic *p*-value may not be applicable criteria for gene selection of our dataset. Hence, for gene ontology analysis, differentially expressed genes were defined as ≥1.5 fold up or down relative to the negative control on the corresponding day. Gene Ontology term enrichment in up and down regulated gene sets were assessed using the GOstat web tool [37]. The GOstat web tool provides a p-value on whether the gene list provided is significantly enriched for genes annotated with a particular Gene Ontology. This is calculated based upon how many genes in the gene set are annotated with the given ontology, and how many genes on the entire microarray (the background) are annotated with the same ontology. We defined the background as the set of genes detected in at least one of the array experiments.

### Antibodies and immunoblotting analysis

Antibodies to the α-tubulin (Sigma) and CDK6 (Santa-Cruz Biotechnology, Santa-Cruz, CA) were used. Cultured cells (5.0×10ˆ5 cells/well) were grown on 6-well plate wells and transfected with miRNAs. 24 hours after the transfection, the cells were lysed and subjected to SDS-polyacrylamide gel electrophoresis. The separated proteins were transferred onto Immobilon membrane (Millipore, Billerica, MA) by electroblotting. Immune complexes were detected by enhanced chemiluminescence (Perkin Elmer) and visualized with LAS 3000 image analyzer (Fuji film, Tokyo, Japan).

## Supporting Information

Table S1 (0.03 MB XLS)

Table S2 (0.27 MB XLS)
